# Classifying Sex from MSCT-Derived 3D Mandibular Models Using an Adapted PointNet++ Deep Learning Approach in a Croatian Population

**DOI:** 10.3390/jimaging11100328

**Published:** 2025-09-24

**Authors:** Eva Shimkus, Ivana Kružić, Saša Mladenović, Iva Perić, Marija Jurić Gunjača, Tade Tadić, Krešimir Dolić, Šimun Anđelinović, Željana Bašić, Ivan Jerković

**Affiliations:** 1 Program in Biochemistry and Biophysics, Amherst College, Amherst, MA 01002, USA; eshimkus27@amherst.edu; 2 Faculty of Forensic Sciences, University of Split, 21000 Split, Croatia; ikruzic@forenzika.unist.hr (I.K.); ijerkovic@unist.hr (I.J.); 3 Faculty of Science, University of Split, 21000 Split, Croatia; sasa.mladenovic@pmfst.hr; 4 Department of Abdominal Surgery, University Hospital Split, 21000 Split, Croatia; iva.peric1@yahoo.com (I.P.); maajur@gmail.com (M.J.G.); ttadic@kbsplit.hr (T.T.); kdolic@kbsplit.hr (K.D.); sandelinovic@kbsplit.hr (Š.A.); 5 Faculty of Health Sciences, University of Split, 21000 Split, Croatia

**Keywords:** artificial intelligence, deep learning, PointNet++, sex estimation, mandible, Croatian population, Gradio app

## Abstract

Accurate sex estimation is critical in forensic anthropology for developing biological profiles, with the mandible serving as a valuable alternative when crania or pelvic bones are unavailable. This study aims to enhance mandibular sex estimation using deep learning on 3D models in a southern Croatian population. A dataset of 254 MSCT-derived 3D mandibular models (127 male, 127 female) was processed to generate 4096-point clouds, analyzed using an adapted PointNet++ architecture. The dataset was split into training (60%), validation (20%), and test (20%) sets. Unsupervised analysis employed an autoencoder with t-SNE visualization, while supervised classification used logistic regression on extracted features, evaluated by accuracy, sensitivity, specificity, PPV, NPV, and MCC. The model achieved 93% cross-validation accuracy and 92% test set accuracy, with saliency maps highlighting key sexually dimorphic regions like the chin, gonial, and condylar areas. A user-friendly Gradio web application was developed for real-time sex classification from STL files, enhancing forensic applicability. This approach outperformed traditional mandibular sex estimation methods and could have potential as a robust, automated tool for forensic practice, broader population studies and integration with diverse 3D data sources.

## 1. Introduction

In forensic anthropology, sex estimation is a crucial element in developing biological profiles of unknown individuals, with knowledge of sex being a prerequisite for the estimation of related factors, including age, stature, and ancestry [1,2,3].

Crania and pelvic bones are most commonly used to evaluate sex [1]; however, other bones with sexually dimorphic features, like the mandible, can be useful tools when crania and pelvic bones are damaged or unavailable [4,5,6]. Traditional methods of sex investigation can be either non-metric (morphologic) or metric. Non-metric methods that visually assess sexually dimorphic traits on the bone of interest based on established standards allow for rapid and cost-efficient sex estimation but can be subjective and can lack reproducibility [7]. In males, mandibular morphological differences include a larger presence of muscle markings, larger tooth size, mandible robustness [8], a broader and taller ramus [8,9,10], gonial eversion [8], and a square chin shape [8,11]; ultimately, however, the accuracy of sex estimation using this approach varies heavily by trait and population.

For example, a study on an American mixed population and a study on a Brazilian mixed population scored ramus shape, finding 94.2% [9] and 57.9% [8] accuracy, respectively, with the Brazilian study concluding that the trait was an unreliable indicator of sex. The Brazilian study also concluded that the shape of the chin was the most sexually dimorphic out of seven analyzed morphological traits (shape of chin, divergence of gonial angle, profile of chin, contour of base of mandible, shape of ramus, profile of ramus, posterior mandibular ramus flexure), with an accuracy of 72.3% in males and 74.0% in females [8]. A Korean population was scored with similar parameters, and researchers found that an accuracy of over 90% could only be attained by simultaneously looking at both the shape of the base of the mandible and the shape of the chin [11]. A study on a German population scored gonial eversion and found 60.3% accuracy overall, with 75.4% for males, 45.2% for females [10].

In a less subjective and more reproducible method of mandibular sex estimation, metric sex evaluation quantifies sexual dimorphisms via the measurement of pinpointed anatomical landmarks. Researchers are able to compare variation in measurements by applying a statistical analysis model such as discriminant function analysis (DFA) or logistic regression (LR). Standard mandibular protocol [12] measures chin height, height of the mandibular body, breadth of mandibular body, bigonial breadth, and bicondylar breadth. A scoping review from Alves et al. [5] comparing 39 works on sex estimation of the mandibular ramus found that these studies primarily utilized the height of the mandibular ramus [13], the angle of the mandible [5], the bicondylar angle [14], and the height of the coronoid process [13] to make sex estimations. The same study reported 23/39 studies that listed mandibular ramus height as the best parameter for sex estimation, with males exhibiting larger heights [5]. However, they note that the accuracy of metric analyses depends heavily on the analysis of multiple measurements in conjunction, rather than the analysis of isolated mandibular features [5].

The accuracy of metric mandibular sex estimation studies also varies by population [5]. Alves et al. noted that in a Brazilian population, bigonial breadth and ramus height examined together gave an overall accuracy of 77.3%, while in a Greek population, the accuracy was 80% [5]. In a northern Indian population, the measurements of coronoid height, projective height, condylar height, and maximum and minimum breadth of ramus combined showed an accuracy of 80.2%, while alone, the coronoid height was the most accurate indicator of sex at 74.1% [13]. In a Romanian population, chin height, bigonial breadth, and bicondylar height combined showed an accuracy of 84%, while bigonial breadth was the most accurate feature (80.5%) when examined alone [14].

These traditional methods, while relatively effective when population differences are considered, often include the risk of observer errors [5,15]. The recent incorporation of neural networks, a subcategory of machine learning, into forensics presents an opportunity to optimize sex estimation to an even higher degree of accuracy and efficiency [2]. Unlike traditional morphometric and morphological approaches to sex estimation, computational neural networks (CNNs) mimic the human brain with statistical models (neurons) that process data and learn patterns through training without direct programming or human supervision [16,17]. CNNs can extract features from a sequence of 2D images, such as slices of computed tomography (CT) images, snapshots of 3D reconstructions, or complete 3D model, capturing morphological details that may not be detectable by human observers or classical statistics [18,19].

Sex evaluation studies applying neural networks to computed tomography datasets for sex estimation achieved impressive accuracy scores [20,21,22,23]. Cao et al. designed a sex estimation CNN model system using a deep learning framework GoogLeNet Inception V4. They tested the system on 1000 2D images obtained from 3D MSCT reconstructions of the Han Chinese population, achieving accuracies of 98.0%, 98.5%, and 94.0% for sex estimation focusing on the ventral pubis (VP), dorsal pubis (DP), and greater sciatic notch (GSN) areas, respectively [22]. Kondou et al. applied DenseNet121 from the MONAI library for feature extraction of 2041 3D skull images reconstructed from postmortem head CT scans. Focusing on skull shape, mandibular roundedness, and cranial ruggedness as sexually dimorphic features, this study on East Asian cadavers achieved an overall accuracy of 93% for sex estimation [23]. Bewes et al. trained sex classification model on 900 2D images of skulls using the GoogLeNet CNN in an Australian population, achieving 95.0% accuracy [20]. A recent study on 3D models obtained from MSCT images showed the potential of applying the described approach on less dimorphic skeletal elements like hyoid bones and more realistic sample sizes, reaching 88–89% of correct sex classifications when using an adapted version of PointNet++ [24].

While many studies have estimated sex using the mandible, few studies globally have investigated deep learning approaches to mandibular sex estimation. Thus, this study aims to utilize 3D segmented mandibular scans derived from multislice computed tomography (MSCT) images of the southern Croatian population to create an automated sex estimation model system using CNNs.

## 2. Materials and Methods

### 2.1. Data Structure and Preparation

This retrospective study used clinical images collected between 2022–2025 from MSCT scans from the University Hospital of Split (UHC). The dataset in this study consisted of 254 mandible and cranial samples (127 M, 127 F). The anonymized MSCT scans of the head and neck were stored in the digital imaging and communications in medicine (DICOM) format. These DICOM files were uploaded to 3D Slicer (v.5.8.0, Kitware Inc., Carrboro, NC, USA). There, the images were cropped by reducing the region of interest (ROI) size to only encompass the head of the patient. This was done to minimize the images’ GPU memory strain on our system and to maximize efficiency. In 3D Slicer, we used the Medical Open Network for AI, MONAI (v.1.3.0, NVIDIA Corporation, Santa Clara, CA, USA) Auto3DSeg extension to define and segment relevant features using the Whole Head Segmentation protocol (v.1.0.1). Computation time on GPU was on average 2 min per CT scan. In Segment Editor module, we refined our anatomical search to exclude soft tissue, only displaying the mandible. Segmented 3D mandibular scans were saved in STL format.

Once STL files of the mandible scans were obtained, they were imported to Blender (v.4.4, Blender Foundation, Amsterdam, The Netherlands), an open-source 3D creation suite with a mesh structure editing feature of vertices. Because some images contained artifacts, they were cropped and cleaned in edit and sculpt modes for elements that were irrelevant to or hindered identification.

**Figure 1:**
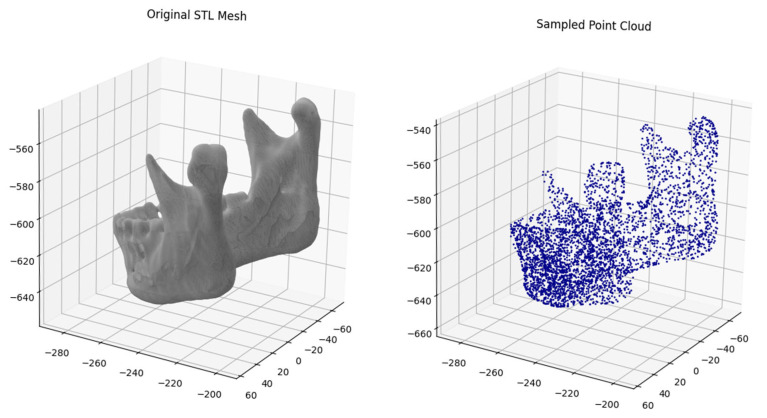
Example of transformation of original stl meshes into 4096 point clouds.

Three-dimensional mandibular models were processed using a Python script locally in Visual Studio Code (v.1.102.0). The script loads STL files from an input directory and samples each mesh to generate 4096 points using uniform surface sampling, without geometry preprocessing (Figure 1). The selection of the 4096-point resolution was based on prior experiments that considered both lower (2048) and higher (8192) resolutions, as the 4096-point resolution provided optimal classification performance (Supplementary Table S1). Sampled points were saved as NumPy arrays (.npy files; NumPy v.2.1.3) in an output directory. The resulting folder with point cloud files was subsequently copied to Google Drive for further analysis using Google Colab.

### 2.2. Dataset Splitting

The dataset consisted of 3D mandibular bone models with filenames prefixed by ‘M_’ for male and ‘F_’ for female samples, including age information. The dataset was balanced to include equal numbers of male and female samples, and split into training (≈60%), validation (≈20%), and test (≈20%) sets using an interleaved assignment strategy. Samples were sorted by age and allocated such that the first three out of every five were assigned to training, the fourth to validation, and the fifth to test, with any remainder added to training, implemented with a fixed random seed for reproducibility. Labels (0 for male, 1 for female) were assigned based on filename prefixes and reassigned after shuffling to ensure consistency. The composition and age descriptives for each split are shown in Table 1.

### 2.3. Models’ Global Architecture

**Figure 2:**
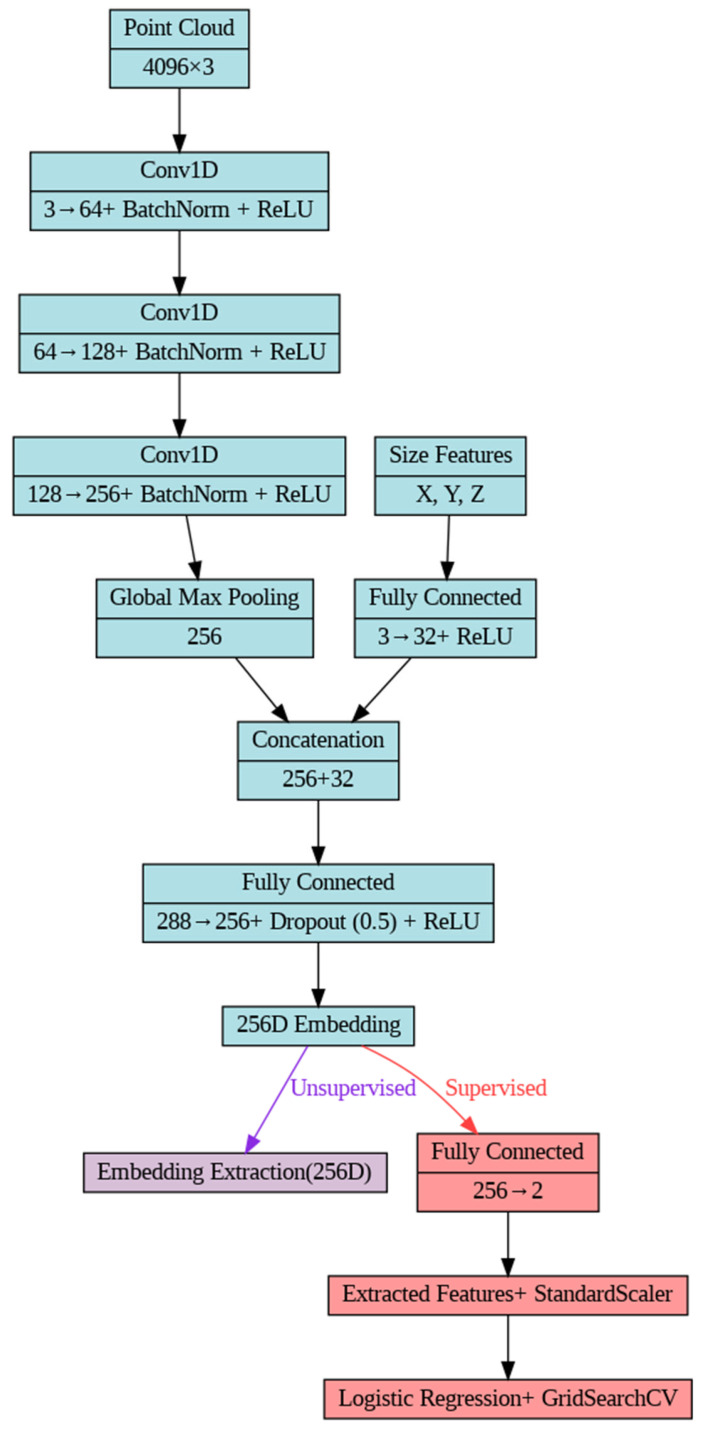
Adapted PointNet++ architecture, illustrating the shared encoder (blue) with divergent unsupervised embedding extraction (purple) and supervised classification (red) paths.

The study employed an adapted PointNet++ architecture for both unsupervised and supervised analyses of 3D mandibular bone models (Figure 2), based on a prior approach implemented for hyoid bone analysis [24] and ablation study (Supplementary Table S1). The architecture consisted of three 1D convolutional layers (mapping 3 to 64, 64 to 128, and 128 to 256 dimensions, each with kernel size 1), incorporating batch normalization and ReLU activation. Global geometric properties were integrated using adaptive max pooling and size features, calculated as the extents of point cloud coordinates and processed through a fully connected layer (3 to 32 dimensions). The resulting 288-dimensional feature vector was combined and passed through a fully connected layer (288 to 256 dimensions) with 50% dropout and ReLU activation, which produced a 256-dimensional embedding. The model was implemented in Python (v.3.11.9) using PyTorch (v. 2.6.0+cu124), with training conducted for 20 epochs (unsupervised) or 50 epochs (supervised), a learning rate of 0.001 (unsupervised) or 0.0003 (supervised), and early stopping (patience 3 or 5) based on validation loss.

### 2.4. Unsupervised Analysis

For unsupervised analysis, the adapted PointNet++ served as the encoder within an autoencoder framework. The decoder comprised two fully connected layers (256 to 512, 512 to 1024) and a final layer (1024 to 4096 × 3) to reconstruct the 4096-point input cloud, optimized using the Chamfer distance loss. Data augmentation included random rotations (up to ±5 degrees per axis) and minor noise (normal distribution, σ = 0.005) applied during training. The 256-dimensional embeddings were extracted from the test set and visualized with t-SNE.

### 2.5. Supervised Analysis

For supervised classification, a classification head (256 to 2 dimensions) was added to the encoder, trained with cross-entropy loss. Data augmentation involved random rotations (up to ±5 degrees per axis) and noise during training. Features were extracted, scaled using StandardScaler, and classified with Logistic Regression, optimized via grid search over hyperparameters (C in [0.1, 0.5, 1, 5, 10], max_iter in [500, 1000]) using five-fold cross-validation. Logistic regression was chosen for its simplicity, direct probability outputs suitable for forensic applications, and good performance given the available sample size. Performance was evaluated using accuracy, sensitivity, specificity, positive predictive value (PPV), negative predictive value (NPV), and Matthews Correlation Coefficient (MCC), with male (label 0) defined as the negative class and female (label 1) as the positive class. These metrics were computed across cross-validation folds, the test set, with confusion matrices plotted to visualize classification outcomes.

### 2.6. Visualizations and Interpretability Tools

For unsupervised analysis, visualization of the 256-dimensional embeddings was performed using t-distributed Stochastic Neighbor Embedding (t-SNE) with a fixed random seed for reproducibility. The reduced two-dimensional embeddings were used to identify extreme samples (minimum and maximum along each t-SNE axis) and a central specimen (based on Euclidean distance from the mean). Projections of the original 4096-point clouds for these selected specimens were generated across XY, XZ, and YZ planes, with fixed axis ranges to standardize visual comparison. Although axes in the t-SNE plots are arbitrary due to the stochastic nature of the algorithm and cannot represent specific anatomical dimensions, they were used to illustrate the relative natural clustering of mandibular specimens by sex.

For supervised analysis, interpretability was assessed using saliency maps derived from the adapted PointNet++ model. Saliency was computed by calculating the gradient norm of the input point cloud with respect to the predicted class probability via backpropagation through the final classification layer. Correctly classified male and female samples, as well as specifically identified misclassified samples, were selected for analysis. Two-dimensional projections (XY, XZ, YZ) of their point clouds were generated, colored by saliency magnitude using a viridis colormap, with normalization based on the 5th and 99th percentiles across all selected samples.

### 2.7. Gradio Application Development

A web-based application for interactive model usage was developed using Gradio, deployed as a Hugging Face Space. The development process involved installing the huggingface_hub library and authenticating with a personal access token to create a new Space repository under a specified username. The Space was initialized with the Gradio SDK, and a local directory was cloned to host the application files. The app.py script was written to integrate the pre-trained PointNet++ model, a pre-fitted StandardScaler, and a trained Logistic Regression classifier, all loaded from saved files. The application accepted STL file uploads, processed them by sampling 4096 points using the trimesh library, centering the points, computing size features, and generating embeddings with the model. These embeddings were scaled and classified using a logistic regression model to predict sex (male or female) with associated probabilities. A 3D scatter plot of the sampled point cloud was generated using Matplotlib (v.3.10.0) for visual preview. Required dependencies, including torch, numpy, scikit-learn, matplotlib, gradio, and trimesh, were listed in a requirements.txt file. Model weights, scaler, and classifier files were copied to the local directory, and the repository was pushed to the Hugging Face Space using the Repository class.

### 2.8. Implementation Details

All processing and development began with initial local data preparation conducted using Visual Studio Code (v.1.102.0) on a Windows 10 (64-bit) system, utilizing a 20-core CPU with 31.63 GB RAM and 952.93 GB total disk storage, with no GPU usage required, executed in Python (v.3.11.9). Other analyses and model constructions were conducted in Google Colab Pro, a cloud-based Python programming environment, as of 14 July 2025, with computational resources including a multi-core CPU and an NVIDIA A100 GPU, executed in Python (v.3.11.13). The workflow utilized key libraries: torch (v.2.6.0+cu124), numpy (v.2.0.2), trimesh (v.4.7.0), scikit-learn (v.1.6.1), matplotlib (v.3.10.0), gradio (v.5.31.0), huggingface_hub (v.0.33.2), scipy (v.1.15.3), tqdm (v.4.67.1), and standard library modules multiprocessing, warnings, pickle, random, and os.

## 3. Results

### 3.1. Unsupervised Analysis

**Figure 3:**
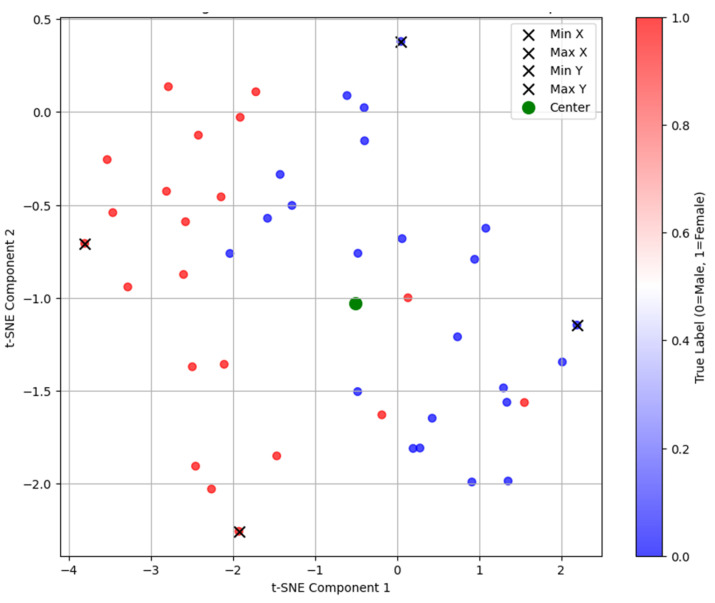
t-SNE projection of embeddings from the unsupervised autoencoder, showing the distribution of male (blue dots) and female (red dots) mandibular specimens.

**Figure 4:**
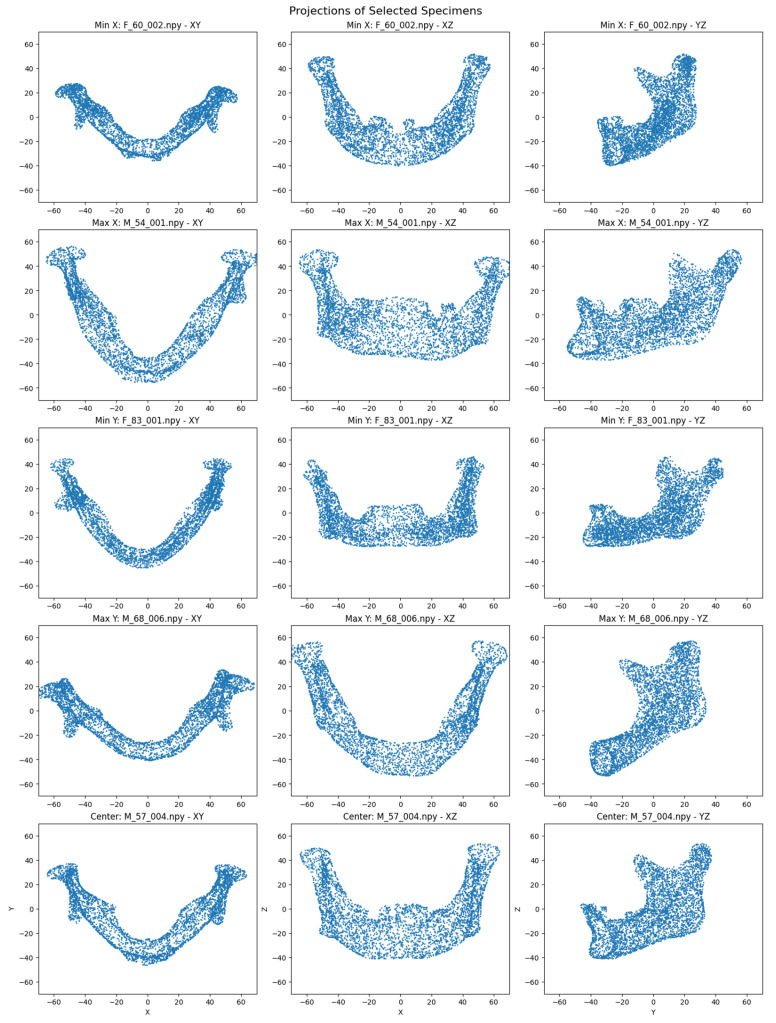
2D projections (XY, XZ, YZ) of point clouds for the minimum and maximum X/Y specimens and the central specimen, derived from t-SNE embeddings.

The autoencoder training stopped early after four epochs due to an increase in validation loss. A t-SNE projection of the embeddings showed male and female specimens in separate regions of the latent space (Figure 3). Visual differences in specimen projections highlight variations in point cloud distributions, which may correspond to sex-specific mandibular features (Figure 4). Specifically, on the x-axis, the mandibles clustered by shape that made them appear shorter (minimum values) or longer (maximum values), while, on the y-axis, they clustered by width that increased from the bottom to the top of the plot.

### 3.2. Supervised Analysis

**Figure 5:**
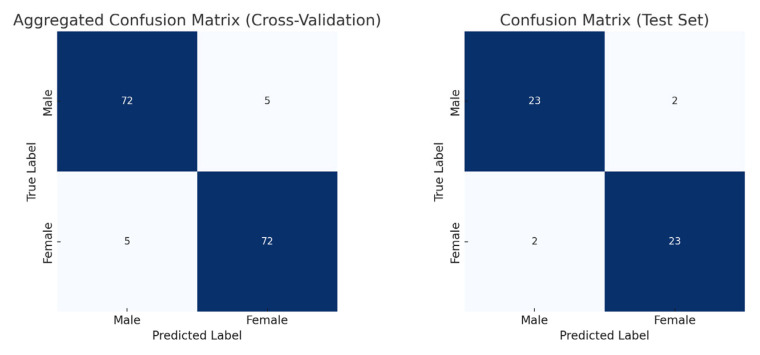
Confusion matrices for aggregated cross-validation and test set.

**Figure 6:**
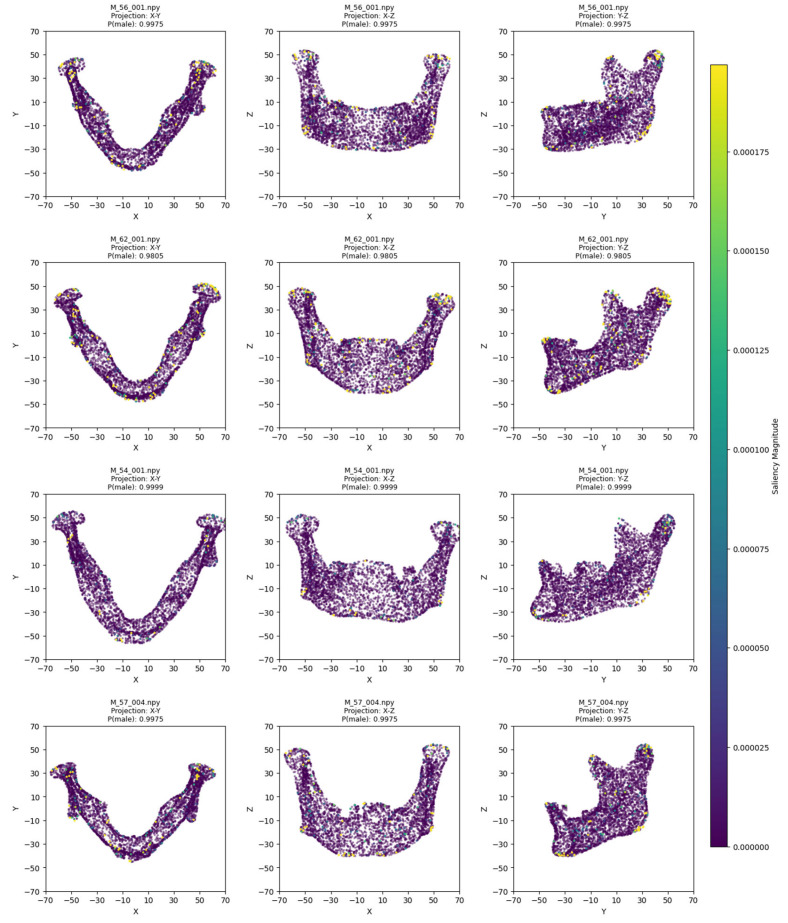
Saliency map projections (XY, XZ, YZ) for four correctly classified males, with color intensity reflecting saliency magnitude.

**Figure 7:**
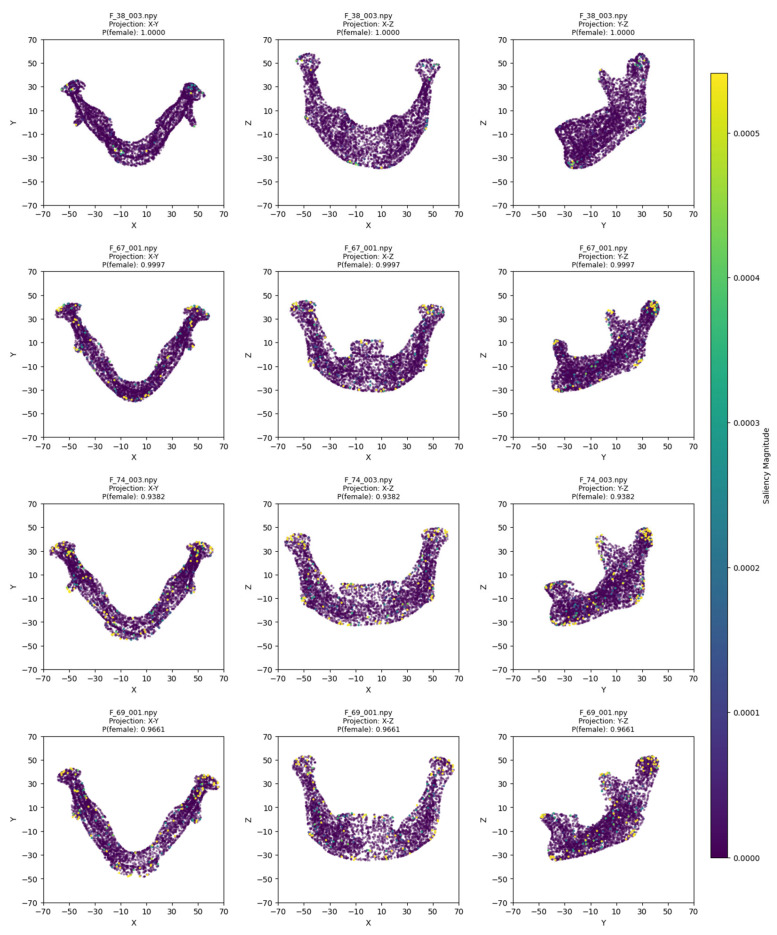
Saliency map projections (XY, XZ, YZ) for four correctly classified females, with color intensity reflecting saliency magnitude.

**Figure 8:**
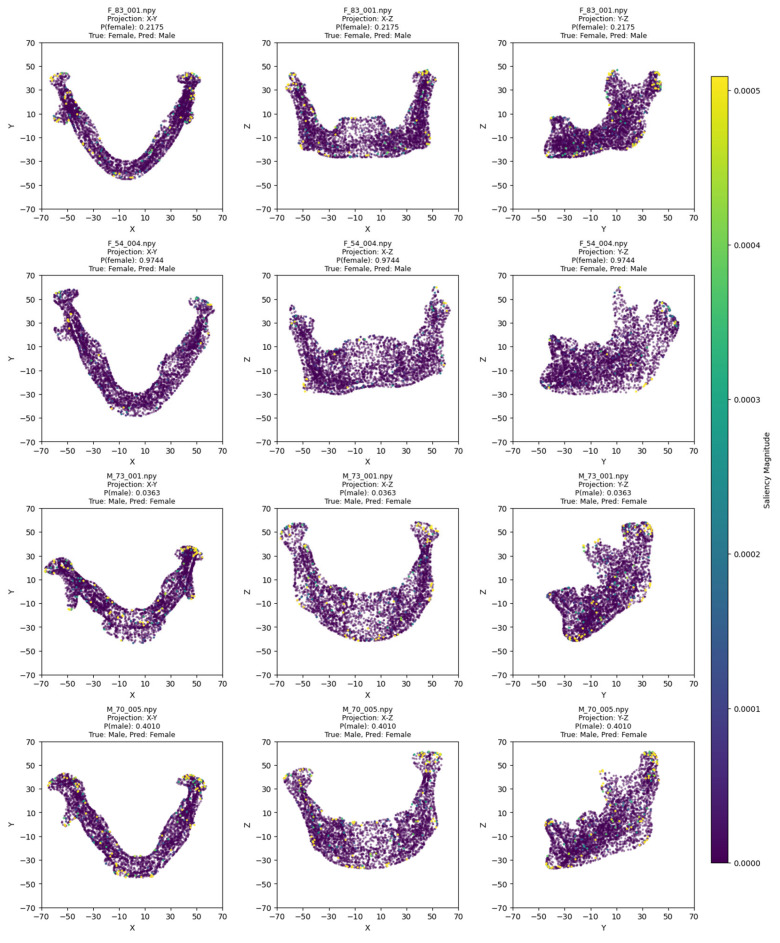
Saliency map projections (XY, XZ, YZ) for the four misclassified mandibular specimens from the test set, with color intensity reflecting saliency magnitude.

The model training ran for 28 epochs with early stopping, recording training losses of 0.78 at epoch 10 and 0.45 at epoch 20. The best Logistic Regression parameters were C = 0.5 and max_iter = 500, optimized for MCC, achieving an average cross-validation accuracy of 0.93 and test set accuracy of 0.92 (Table 2). Low cross-validation accuracy variance (SD = 0.05) across folds, combined with consistent 93% CV/92% test accuracy, suggests no overfitting. Confusion matrices for the aggregated cross-validation and test set (Figure 5) showed balanced error distributions, with 10 total misclassifications across five folds and 4 in the test set. Figure 6 and Figure 7 show saliency map projections for four correctly classified male and four correctly classified female mandibular specimens, with colors indicating saliency magnitude from low (dark) to high (bright). Figure 8 displays saliency map projections for the four misclassified test set specimens, also colored by saliency magnitude.

The most contributing mandibular regions for sex classification were the chin, gonial, condylar, and coronoid parts. On the x-y projection, male mandibles demonstrated greater length of the body and the whole bone. This was evident in all examples except for M_57_004, which lacked such length but was not misclassified, probably due to pronounced masculine traits on other axes. For instance, in the x-z projection, the most distinguishing factor was the chin shape, which was more squared and wider in male specimens and more rounded in female mandibles. Additionally, the presence of a gonial eversion was evident in all males, not only on the x-z axis, but also on the y-z axis. On that axis, male mandibles demonstrated larger body height and ramal breadth. One of the elements distinguishable in all projections was also the condylar processes, which showed greater robusticity in male individuals.

The misclassified female mandibles (Figure 8) had greater overall size (especially length), body height, and ramus width than correctly classified females (Figure 7). They also showed very pronounced gonial eversion, antegonial notches, and rectangular chins. On the other hand, misclassified male specimens resembled correctly classified female specimens in most size features and also had less squared chins.

### 3.3. Supervised Analysis

**Figure 9:**
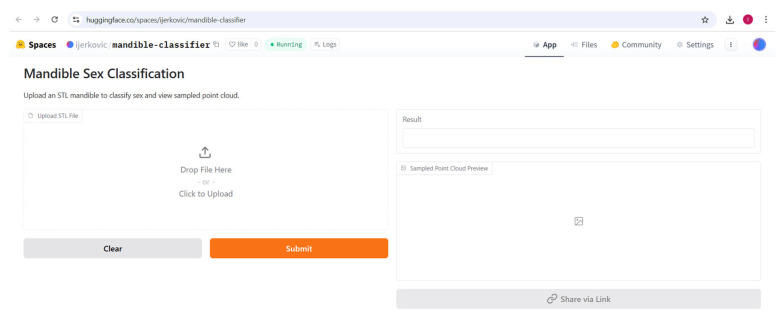
Gradio app for sex classification using mandibular 3D models deployed at Hugging Face.

**Figure 10:**
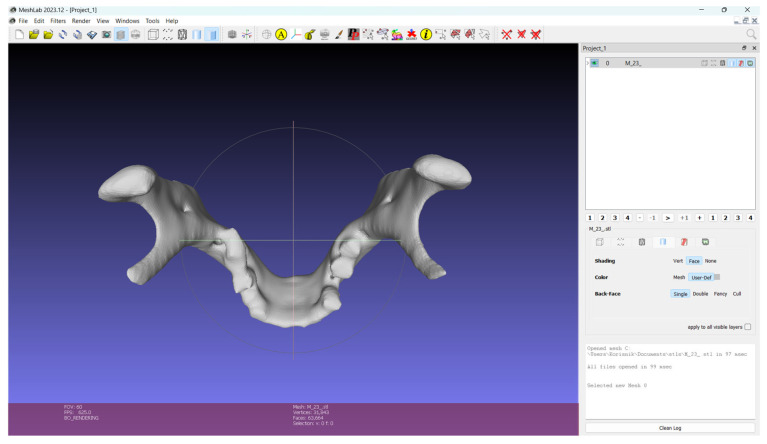
Male mandible in .stl format used for app demonstration.

**Figure 11:**
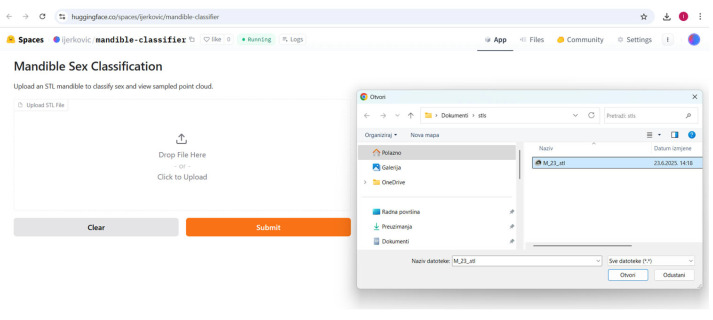
The process of loading .stl files.

**Figure 12:**
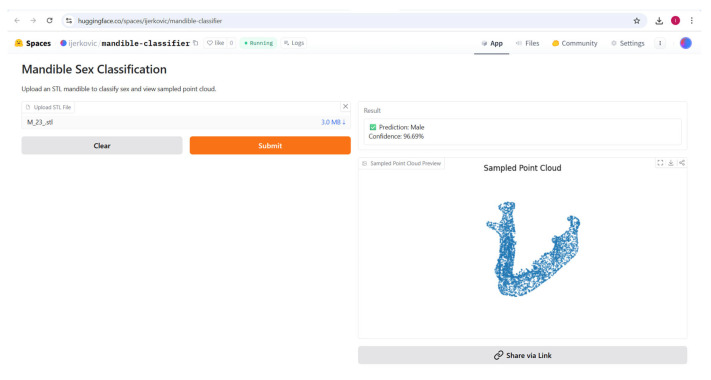
The classified specimen, including predicted class, probability, and a preview of the sampled point cloud.

The Gradio app was deployed to the Hugging Face Space at https://huggingface.co/spaces/ijerkovic/mandible-classifier (accessed on 14 June 2025). The app, which accepts an uploaded STL file, produces a prediction (e.g., “Male” or “Female”) along with a confidence percentage and a 3D point cloud preview (Figure 9, Figure 10, Figure 11 and Figure 12). Please note that the app may enter sleep mode during periods of inactivity; users experiencing access issues are advised to contact the corresponding author.

### 3.4. Comparative Analysis of State-of-the-Art Methods

To contextualize the performance of our model, we compared it with results from recent deep learning approaches applied to other skeletal elements (Table 3). Our adapted PointNet++ achieved 93% cross-validation accuracy and 92% test set accuracy, exceeding previous mandible- and hyoid-based studies, and reaching a level comparable to skull- and pelvis-based CNN frameworks, which are traditionally considered the most dimorphic bones for sex estimation.

## 4. Discussion

This study developed and validated an adapted PointNet++ deep learning model for sex estimation using 3D mandibular models derived from MSCT scans in a southern Croatian population, achieving accuracies of 93% in cross-validation and 92% on an independent test set. A wide age range, including a considerable number of edentulous elders, did not hinder the model’s performance but rather made it even more adapted to real-life scenarios, where ante- or postmortem tooth loss is a frequent finding. The results were supplemented with a web application that can further be used to test the method. This approach encourages the shift from proof-of-concept to applicable biological profiling methods based on deep learning.

The high performance of our model can be attributed to the PointNet++ architecture’s effectiveness in processing raw 3D point clouds, which capture subtle geometric variations without manual interventions. Saliency maps highlighted regions like the chin (more squared in males), the gonial region (everted in males with pronounced antegonial notches), and the superior ramal structures (wider in males), which are traditionally recognized as sexually dimorphic [9,30,31]. Misclassifications primarily occurred in specimens exhibiting opposite-sex features, such as squared female or rounded male chins. The unsupervised t-SNE projections further supported this, revealing clustering based on size and similar morphological features. Although we did not compute manual landmark-based measurements in this study or conducted visual scoring, these parallels illustrate that the model’s focus overlaps with classical forensic parameters. This approach maintains interpretability at the level of expert visual assessment, while avoiding additional manual preprocessing steps.

Compared to traditional morphological and metric methods, our approach demonstrated superior accuracy. Non-metric assessments of mandibular traits, such as ramus shape or chin profile, typically yield accuracies ranging from 59% to 94%, with considerable variation by population and trait [8,9,10,11]. Metric studies, typically applying DFA or logistic regression on mandibular traits like ramus height or bigonial breadth, report accuracies around 77–84%, with best results when multiple characteristics are combined [5]. Still, even with multiple measurements, the mandible often performs below the skull, pelvis, and long bones, as shown in Spradley and Jantz [32]. Our model, with an accuracy of 92–93%, generally surpasses these, and by implementing a fully automated approach, it eliminates the impact of subjectivity and observer errors.

In the context of deep learning applications for sex estimation in forensic anthropology, our results are competitive given the anatomical region studied. Studies on more sexually dimorphic elements, such as the pelvis, have achieved higher accuracies (94–98%), as demonstrated by Cao et al. [22], who employed GoogLeNet Inception V4 on 2D images extracted from 3D MSCT reconstructions cropped to comprise specific pelvic regions (ventral pubis, dorsal pubis, and greater sciatic notch). Similarly, skull-based models report 93–97%. Kondou et al. [23] applied a 3D gated attention-based MIL framework with DenseNet121 from the MONAI library to postmortem CT skull reconstructions of 2041 East Asian cadavers, achieving 93% accuracy using volumetric input focused on whole-skull morphology, particularly mandibular roundedness and overall shape. Lye et al. [26] used 3D cranial CT data from 200 Indonesian individuals and trained a ResNet-based model to estimate sex, with Walker traits integrated into the model through multi-task learning to enhance performance, reaching 97% accuracy. A respectable accuracy rate was also achieved for 2D images of the humerus (91%) and clavicle (88.33–95%) using ResNet50 and GoogLeNet, respectively [28,29]. For 2D mandible images and various CNN architectures on 200 Anatolian samples, the best-performing model reached an accuracy of 88% (LeNet5_bn_do_skipcon), though with a notable imbalance between sensitivity and specificity (a 23% difference) [25].

The accuracy of our mandible-based study falls within the upper range reported in related studies (see Table 3), despite the bone’s moderate dimorphism, and outperforms a prior PointNet++ application on hyoid bones (88–89%) in the same population [24]. This confirms that 3D point cloud processing enhances feature extraction for less dimorphic elements, even with relatively modest datasets. Additionally, the model’s performance was not compromised despite the inclusion of a wide age range (18–93 years) and edentulous individuals who were previously shown to alter sexual dimorphism patterns [33].

The implications of this work extend to forensic practice, where mandibles are often durable and well-preserved. The Gradio app offers a user-friendly tool for rapid sex estimation, requiring only STL file uploads and providing probabilities and visualizations. This bridges the gap between concept and application, supporting practical biological profiling in mass disasters or for unidentified remains. Population-specificity (southern Croatian) ensures relevance to regional forensics, though cross-population validation is warranted given ancestral variations in mandibular size and morphology. Although a user-friendly Gradio interface was developed to demonstrate applicability, the method itself remains in the validation stage. At present, its reliability outside the studied dataset has not been established, and independent replication on diverse populations and imaging protocols will be essential before any casework application can be recommended. This does not diminish the novelty of our approach but reflects the necessary caution when introducing AI-based methods into forensic anthropology. Until such external validation is achieved, the tool should be regarded as an experimental aid for further research rather than a substitute for expert evaluation.

While the model was validated against ground-truth sex labels from clinical documentation, no independent validation by forensic practitioners or clinicians was conducted at this stage. Such expert testing, alongside replication on external datasets, will be an important next step before routine forensic application. Future studies should also prioritize expanding datasets both in size and across diverse populations, as broader representation will improve generalizability and help calibrate performance parameters. While we did not record dental status explicitly for each specimen, the balanced age distribution across training, validation, and test sets makes it reasonable to assume that edentulous mandibles were similarly distributed. However, explicit evaluation of covariates such as age and dental status may further refine classification and interpretability. Additional improvements could be achieved through advanced augmentation strategies and systematic benchmarking against alternative architectures to validate the advantages of adapted PointNet++. For full applicability in forensic practice, models should also be adapted to accept mesh data from different acquisition sources, including 3D surface scanners and everyday devices such as cellphones equipped with LIDAR, to assess their feasibility for in situ application. Combining saliency-derived features with traditional quantitative anthropometric measures may additionally strengthen the bridge between deep learning outputs and established forensic protocols. Finally, external validation on independent multicenter cohorts will be essential to confirm robustness across populations, imaging protocols, and real-world forensic casework.

## Figures and Tables

**Table 1:** Demographic composition of the sample used in the study.

Split	Total	Males	Females	Age Mean	Age Median	Age Min	Age Max	Age Std
Overall	254	127	127	58.59	62.50	18.00	93.00	16.35
Training	154	77	77	58.42	62.00	18.00	93.00	16.66
Validation	50	25	25	58.60	62.00	20.00	84.00	15.93
Test	50	25	25	59.14	63.00	21.00	85.00	15.81

**Table 2:** Performance of the supervised classification model on average cross-validation and test set.

Metric	Cross-Validation (Avg)	Test Set
Accuracy	0.93	0.92
Sensitivity	0.93	0.92
Specificity	0.94	0.92
PPV	0.93	0.92
NPV	0.94	0.92
MCC	0.87	0.84

**Table 3:** Reported performances of deep learning models for sex estimation from different skeletal elements, compared with the present study.

Study (Year)	Skeletal Element	Dataset/Population	Method	Reported Performances
This study (2025)	Mandible (3D MSCT, Croatian sample)	254 mandibles	Adapted PointNet++ (point clouds + LR)	93% (CV)/92% (test)
Kuha et al. (2024) [25]	Mandible (2D photographs, South African sample)	193 mandibles	CNN variants (best: LeNet5_bn_do_skipcon)	Acc: 84% (val)/88% (test)
Bewes et al. (2019) [20]	Skull (2D lateral CT reconstructions)	1000 skulls (Adelaide, Australia)	GoogLeNet CNN (transfer learning)	Acc: 95% (test)
Kondou et al. (2023) [23]	Skull (3D PMCT, East Asian cadavers)	1234 skulls (Japan)	DenseNet121 with gated MIL	Acc: 93% (test)
Lye et al. (2024) [26]	Skull (3D CT, Indonesian sample)	200 skulls (Indonesia)	Multi-task CNN (sex + Walker traits)	Acc: 97% (test)
Noel et al. (2024) [27]	Skull (3D CT, Cedars-Sinai, USA)	98 skulls (50M, 48F; multi-ethnic)	ResNet3D, PointNet++, MeshNet (DL comparison)	All AUC > 0.9; PointNet++ highest
Cao et al. (2021) [22]	Pelvis (VP, DP, GSN regions, 3D CT + surface scans)	1000 CT pelvises + 105 scanned	GoogLeNet Inception V4 CNN	Acc (test): 94–98% (CT)/97.1% (scans)
Jerković et al. (2025) [24]	Hyoid (3D MSCT, Croatian sample)	202 hyoids	Adapted PointNet++ (point clouds + SVM)	Acc: 88.3% (CV)/88.7% (test)
Venema et al. (2023) [28]	Humerus (distal epiphysis, Mediterranean sample)	417 humeri	ResNet50 (transfer learning + Grad-CAM)	
				Acc: 87.8 (val)/91.0% (test)
Pichetpan et al. (2023) [29]	Clavicle (photographs, Thai sample)	200 clavicles	GoogLeNet CNN (multi-view)	Acc: 88.3–95% (validation)

## Data Availability

The original contributions presented in this study are included in the article/Supplementary Materials. Further inquiries can be directed to the corresponding author.

## Supplementary Materials

The following supporting information can be downloaded at: https://www.mdpi.com/article/10.3390/jimaging11100328/s1, Table S1: Comparative results of ablation experiments across model architectures and point cloud sizes (5-fold cross-validation). They include point cloud data for mandibles in .npy files, train/validation/test split definitions in .pkl files, and Python scripts and Jupyter notebooks for the classification pipeline using EnhancedPointNet++ and Logistic Regression.

## Abbreviations

The following abbreviations are used in this manuscript:

Abbreviation	Full Term
CNN	Computational Neural Network
CT	Computed Tomography
DFA	Discriminant Function Analysis
DICOM	Digital Imaging and Communications in Medicine
DP	Dorsal Pubis
GSN	Greater Sciatic Notch
LR	Logistic Regression
MCC	Matthews Correlation Coefficient
MONAI	Medical Open Network for Artificial Intelligence
MSCT	Multislice Computed Tomography
NPV	Negative Predictive Value
PPV	Positive Predictive Value
ROI	Region of Interest
STL	Stereolithography
t-SNE	t-distributed Stochastic Neighbor Embedding
UHC	University Hospital of Split
VP	Ventral Pubis

## References

[1] Krogman W.M., Charles C. (2013). The Human Skeleton in Forensic Medicine.

[2] Wang X., Liu G., Wu Q., Zheng Y., Song F., Li Y. (2024). Sex Estimation Techniques Based on Skulls in Forensic Anthropology: A Scoping Review. PLoS ONE.

[3] DiGangi E.A., Moore M.K. (2013). Research Methods in Human Skeletal Biology.

[4] Hazari P., Hazari R.S., Mishra S., Agrawal S., Yadav M. (2016). Is There Enough Evidence so That Mandible Can Be Used as a Tool for Sex Dimorphism? A Systematic Review. J. Forensic Dent. Sci..

[5] Alves N., Ceballos F., Muñoz L., Figueiredo Deana N. (2022). Sex Estimation by Metric Analysis of the Angle of Mandible and the Mandibular Ramus: A Systematic Review. Int. J. Morphol..

[6] Toneva D., Nikolova S., Agre G., Zlatareva D., Fileva N., Lazarov N. (2024). Sex Estimation Based on Mandibular Measurements. Anthropol. Anz..

[7] Lewis C.J., Garvin H.M. (2016). Reliability of the Walker Cranial Nonmetric Method and Implications for Sex Estimation. J. Forensic Sci..

[8] Deana N.F., Alves N. (2017). Nonmetrical Sexual Dimorphism in Mandibles of Brazilian Individuals. Biomed. Res..

[9] Loth S.R., Henneberg M. (1996). Mandibular Ramus Flexure: A New Morphologic Indicator of Sexual Dimorphism in the Human Skeleton. Am. J. Phys. Anthropol..

[10] Kemkes-Grottenthaler A., Löbig F., Stock F. (2002). Mandibular Ramus Flexure and Gonial Eversion as Morphologic Indicators of Sex. HOMO.

[11] Hu K., Koh K., Han S., Shin K., Kim H. (2006). Sex Determination Using Nonmetric Characteristics of the Mandible in Koreans. J. Forensic Sci..

[12] Langley N.R., Jantz L.M., McNulty S., Maijanen H., Ousley S.D., Jantz R.L. (2016). Data Collection Procedures for Forensic Skeletal Material 2.0.

[13] Saini V., Srivastava R., Rai R.K., Shamal S.N., Singh T.B., Tripathi S.K. (2011). Mandibular Ramus: An Indicator for Sex in Fragmentary Mandible. J. Forensic Sci..

[14] Marinescu M., Panaitescu V., Rosu M. (2013). Sex Determination in Romanian Mandible Using Discriminant Function Analysis: Comparative Results of a Time-Efficient Method. Rom. J. Leg. Med..

[15] Nikita E., Nikitas P. (2020). On the Use of Machine Learning Algorithms in Forensic Anthropology. Leg. Med..

[16] Bu W.-Q., Guo Y.-X., Zhang D., Du S.-Y., Han M.-Q., Wu Z.-X., Tang Y., Chen T., Guo Y.-C., Meng H.-T. (2023). Automatic Sex Estimation Using Deep Convolutional Neural Network Based on Orthopantomogram Images. Forensic Sci. Int..

[17] Baban M.T.A., Mohammad D.N. (2024). A New Approach for Sex Prediction by Evaluating Mandibular Arch and Canine Dimensions with Machine-Learning Classifiers and Intraoral Scanners (a Retrospective Study). Sci. Rep..

[18] Thurzo A., Kosnáčová H.S., Kurilová V., Kosmeľ S., Beňuš R., Moravanský N., Kováč P., Kuracinová K.M., Palkovič M., Varga I. (2021). Use of Advanced Artificial Intelligence in Forensic Medicine, Forensic Anthropology and Clinical Anatomy. Healthcare.

[19] Dutta S., Keerthana R., Shekar D.C., Kumar S.S. Biological Profile Estimation of the Human Skeleton Based Forensic Identification Using Deep Learning Image Classification. Proceedings of the 5th International Conference on IoT Based Control Networks and Intelligent Systems, ICICNIS 2024.

[20] Bewes J., Low A., Morphett A., Pate F.D., Henneberg M. (2019). Artificial Intelligence for Sex Determination of Skeletal Remains: Application of a Deep Learning Artificial Neural Network to Human Skulls. J. Forensic Leg. Med..

[21] Oner Z., Turan M.K., Oner S., Secgin Y., Sahin B. (2019). Sex Estimation Using Sternum Part Lenghts by Means of Artificial Neural Networks. Forensic Sci. Int..

[22] Cao Y., Ma Y., Vieira D.N., Guo Y., Wang Y., Deng K., Chen Y., Zhang J., Qin Z., Chen F. (2021). A Potential Method for Sex Estimation of Human Skeletons Using Deep Learning and Three-Dimensional Surface Scanning. Int. J. Legal Med..

[23] Kondou H., Morohashi R., Kimura S., Idota N., Matsunari R., Ichioka H., Bandou R., Kawamoto M., Ting D., Ikegaya H. (2023). Artificial Intelligence-Based Forensic Sex Determination of East Asian Cadavers from Skull Morphology. Sci. Rep..

[24] Jerković I., Bašić Ž., Kružić I. (2025). Deep Learning-Based Sex Estimation of 3D Hyoid Bone Models in a Croatian Population Using Adapted PointNet++ Network. Sci. Rep..

[25] Kuha A., Ackermann J., Junno J.A., Oettlé A., Oura P. (2024). Deep Learning in Sex Estimation from Photographed Human Mandible Using the Human Osteological Research Collection. Leg. Med..

[26] Lye R., Min H., Dowling J., Obertová Z., Estai M., Bachtiar N.A., Franklin D. (2024). Deep Learning versus Human Assessors: Forensic Sex Estimation from Three-Dimensional Computed Tomography Scans. Sci. Rep..

[27] Noel L., Fat S.C., Causey J.L., Dong W., Stubblefield J., Szymanski K., Chang J.-H., Wang P.Z., Moore J.H., Ray E. (2024). Sex Classification of 3D Skull Images Using Deep Neural Networks. Sci. Rep..

[28] Venema J., Peula D., Irurita J., Mesejo P. (2023). Employing Deep Learning for Sex Estimation of Adult Individuals Using 2D Images of the Humerus. Neural Comput. Appl..

[29] Pichetpan K., Singsuwan P., Intasuwan P., Sinthubua A., Palee P., Mahakkanukrauh P. (2024). Sex Determination Using the Clavicle by Deep Learning in a Thai Population. Med. Sci. Law.

[30] Krogman W.M., İşcan M.Y., Charles C. (1986). The Human Skeleton in Forensic Medicine.

[31] White T.D., Folkens P.A. (2005). The Human Bone Manual.

[32] Spradley M.K., Jantz R.L. (2011). Sex Estimation in Forensic Anthropology: Skull versus Postcranial Elements. J. Forensic Sci..

[33] Ramphaleng T., Billings B., Hemingway J. (2025). The Effect of Tooth Loss on Sexual Dimorphism of South African Mandible Using Geometric Morphometrics. Forensic Sci. Int. Rep..

