# Supplementary material for: Classifying Sex from MSCT-Derived 3D Mandibular Models Using an Adapted PointNet++ Deep Learning Approach in a Croatian Population
Source: J Imaging. 2025 Sep 24;11(10):328. doi: 10.3390/jimaging11100328 (PMC12565416; doi:10.3390/jimaging11100328)
Supplement: Supplementary file 1 [file jimaging-11-00328-s001.zip › jimaging-3801106-supplementary.pdf]

Supplementary Table 1. Comparative results of ablation experiments across model architectures and point cloud sizes (5-fold cross-validation).

Model type	Points	Acc	Sens	Spec	PPV	NPV	MCC
**Size only (LR)**	2048	0.804	0.814	0.794	0.800	0.821	0.614
	4096	0.809	0.824	0.794	0.801	0.831	0.625
	8192	0.799	0.824	0.774	0.786	0.824	0.604
**Adapted PN++ (end-to-end, no size)**	2048	0.705	0.912	0.499	0.664	0.681	0.428
	4096	0.729	0.872	0.584	0.708	0.663	0.464
	8192	0.720	0.913	0.527	0.683	0.687	0.454
**Adapted PN++ + size (end-to-end)**	2048	0.716	0.737	0.698	0.732	0.768	0.466
	4096	0.721	0.794	0.648	0.710	0.804	0.476
	8192	0.716	0.718	0.718	0.753	0.763	0.474
**Adapted PN++ (LR, no size)**	2048	0.928	0.908	0.948	0.945	0.916	0.858
	4096	0.902	0.897	0.909	0.918	0.911	0.817
	8192	0.916	0.920	0.911	0.915	0.931	0.838
**Adapted PN++ + size (LR)**	2048	0.909	0.883	0.933	0.940	0.898	0.826
	4096	**0.935**	0.934	0.935	0.935	0.937	0.870
	8192	0.922	0.898	0.949	0.950	0.908	0.853

Supplementary Table 1 summarizes a series of ablation experiments designed to evaluate how different model architectures and point cloud resolutions affect performance in sex classification. We compared: (i) logistic regression (LR) using only size features, (ii) end-to-end Adapted PointNet++ without size features, (iii) end-to-end Adapted PointNet++ with concatenated size features, (iv) Adapted PointNet++ feature extractor with LR on combined learned and size features, and (v) Adapted PointNet++ feature extractor with LR on learned features only.

Three point cloud resolutions were tested (2048, 4096, and 8192 points). Results are reported as mean metrics across five folds. The best performing models were LR-based approaches that integrated size features, whereas end-to-end models, particularly without size features, showed weaker and less stable performance.
